# Children’s physical activity during a segmented school week: results from a quasi-experimental education outside the classroom intervention

**DOI:** 10.1186/s12966-017-0534-7

**Published:** 2017-06-20

**Authors:** Mikkel Bo Schneller, Jasper Schipperijn, Glen Nielsen, Peter Bentsen

**Affiliations:** 1 Health Promotion, Steno Diabetes Center Copenhagen, the Capital Region of Denmark, Niels Steensens Vej 6, DK-2820 Gentofte, Denmark; 2 Institute of Sports Science and Clinical Biomechanics, University of Southern Denmark, Campusvej 55, DK-5230 Odense M, Denmark; 3 Department of Nutrition, Exercise and Sports, University of Copenhagen, Nørre Allé 53, DK-2200 Copenhagen N, Denmark

**Keywords:** Movement integration, Physical activity domains, School-based physical activity, Segmented physical activity, TEACHOUT

## Abstract

**Background:**

Movement integration (MI) into traditional classroom teaching is a promising opportunity for children to increase physical activity (PA). Education outside the classroom (EOtC) can be regarded as MI, and has increased children’s PA in case studies. The aim of this study is to investigate the effects of EOtC on children’s PA by segmenting weekly activity-related behavior into a range of day types and domains.

**Methods:**

In a quasi-experimental design, 33 classes were recruited and participants’ PA was objectively measured using accelerometers taped to the lower back. In total, 361 (10.89 ± 1.03 years) participants with 7 days of 24 h wear time per day were included in a day type PA analysis, and 194 of these participants (10.46 ± 0.99 years) provided information on time spent in specific domains (e.g. EOtC or recess) and were included in a domain-specific PA analysis. Differences in proportion of time spent in PA intensities were tested using mixed-effects regression models.

**Results:**

More moderate-to-vigorous physical activity (MVPA) occurred on days with physical education (PE) than days with EOtC (girls 0.79%, *p* = .001, CI = .26% to 1.31%; boys 1.35%, *p* = .003, CI = .32% to 2.38%), while no difference was found between EOtC days and school days without EOtC and PE. Light physical activity (LPA) was higher on EOtC days than school days without EOtC and PE (girls 2.43% *p* < .001, CI = 1.21% to 3.65%; boys 2.08%, *p* < .001, CI = .69% to 3.47%) and PE days (girls 2.18%, *p* < .001, CI = .80% to 3.56%; boys 2.40%, *p* < .001, CI = .83% to 3.96%). Comparing EOtC and classroom domains, boys proportionally spent 7.95% (*p* < .001, CI = 3.00% to 12.90%) more time in MVPA while no difference (*p* = 1.000) was measured for LPA, and girls had no difference (*p* = .176) in MVPA, but spent 9.76% (*p* < .001, CI = 7.12% to 12.41%) more time in LPA.

**Conclusions:**

EOtC was implemented without the provision of additional resources and with positive effects on PA. Findings suggest EOtC as a way to provide children with an additional opportunity to accumulate PA within the existing school setting.

## Background

There is evidence that physical activity (PA) is associated with a reduction in a number of physiological risk factors [1, 2], as well as increased cognitive function [3, 4], academic achievement [5, 6], and mental health [7–9]. Schools as an arena have the potential to transform a portion of the time in which children are inactive into time spent being physically active, which e.g., have led to the development of WHO’s holistic approach framework “Health Promoting Schools” aiming to promote health and well-being of children and adolescents [10]. Schools’ core mandates and main purpose, however, remain children’s academic learning. Therefore, schools face increasing demands for their children to achieve academic goals, which may encourage them to allocate less time for PE and recess and more for curriculum-based classroom activities, a trend seen in the US between 2000 and 2014 [11]. Recess (including lunch break) and PE are typically the domains with the highest proportion of time spent in moderate-to-vigorous physical activity (MVPA) during school hours [12, 13]. Consequently, PA during school hours may decline unless the trend is changed or PA is integrated into curriculum-based activities. School-based PA should therefore preferably come from all domains of a school day rather than only the traditional domains of PE and recess.

Recently, there have been calls for school-based PA interventions targeting the school curriculum and seeking integration with the existing practices within educational systems and schools [14]. Thus, there is a growing need to develop, implement and evaluate educational practices that can integrate PA into school hours in a school setting that is required to reach increasingly higher academic goals. The use of movement integration (MI) into traditional classroom time seems a promising opportunity to increase children’s PA [15].

Education outside the classroom (EOtC) is an example of an ‘add-in’ and holistic school-based PA promotion strategy as it aims to promote, in addition to PA, learning, social relations, motivation, and well-being [16]. EOtC changes the physical classroom to whatever is chosen by the teacher, such as a green area close to the school, and thereby offers teachers the possibility to use different pedagogy and in many cases provides extra space for children to be active [17, 18]. It can therefore be regarded as an educational approach to MI. Case studies investigating the effects of regular EOtC have reported improved physical activity [19, 20], academic learning [21, 22], social relations [23], and well-being [24]. EOtC is now a widely practiced teaching approach in Denmark, where national surveys have shown an increase in the proportion of schools with at least one class regularly using EOtC – from 14% in 2007 (52% response rate among all Danish schools) [25] to 18.4% in 2014 (90% response rate) [26] – with increased provision also reported in Scotland from 2006 to 2014 [27].

Therefore, the aim of this study is to evaluate and investigate how EOtC affects daily PA in a larger sample of school-aged children. Specifically, we compare the proportion of time spent in different PA intensities between different day types and within certain domains specific to both school (i.e. EOtC, classroom activities, PE, and recess) and leisure time (i.e. school days and weekend days), repeated by sex.

## Methods

### Setting and study design

This study is part of the larger quasi-experimental TEACHOUT study [16] set in schools across Denmark, located in both rural and urban areas, with the purpose of evaluating and investigating the influence of regular EOtC on PA, academic learning, motivation for learning, social relations, and well-being among the same group of school-aged children in the third to sixth grades (9–13 years old). In Danish primary and secondary schools, teachers are allowed “freedom of methods” to adhere to curricula targets decided by the Danish Ministry of Education within each subject taught [28]. A new public school reform was implemented across Danish schools in August 2014, which included initiatives such as requiring school staff and children to spend 5.5 to 8.5 h more in school every week, to provide pupils with an average of 45 min of daily PA, for schools to seek more active cooperation with local sports clubs, and for teachers to empower children to more actively engage in the educational activities [28]. In Denmark, children are randomly assigned to a class within the school district where they live at enrolment in grade 0. This means that the demographic characteristics of children in two parallel classes can be expected to be comparable [29]. In the TEACHOUT study, data were collected from children who were sampled into EOtC intervention classes and parallel classes at the same school and grade level, based on the willingness of teachers to participate in the study. As such, approximately half the children from whom PA data were obtained attended a comparison class in which EOtC was not supposed to be a regular curriculum-based activity. All data from participating children were pooled and analysed as the amount of EOtC varied greatly between participating classes, and some control classes had practiced EOtC. Instead of comparing the original intervention and control groups we performed all analyses based on the actual exposure to EOtC by comparing days with and without EOtC, as well as the specific EOtC domain with other domains.

### Education outside the classroom

Curriculum-based educational activities are considered EOtC if they are moved outside the school buildings to either green (e.g. a woodland or a nature area) or cultural (e.g. a library or a museum) settings on a regular basis (at least 1 day fortnightly) [25, 30]. EOtC is typically used for educational activities in primary schools that benefits from a more illustrative and hands on teaching approach, e.g. abstract concepts and skills, with a strong tradition for using green or natural spaces as location. Ways to perform an EOtC session could for example include calculating the surface area of the school soccer field, drafting a text about a forest while being in one, and learning about the historic significance of a place while visiting it [18]. The main characteristic of all EOtC activities is the change of setting, which can provide teachers with the possibility to use different pedagogical approaches and thereby include more PA, stimulate senses, conduct practical experiments, play, problem solving, and cooperation [16]. The primary aim of EOtC is to facilitate children’s learning processes by providing a motivating setting that differs from the traditional classroom. Increased PA is a potential secondary outcome or perhaps a means to achieve the teaching aim. The TEACHOUT study design and rationale can be found in the study protocol paper [16] and more in-depth information on EOtC activities in Denmark can be found in an inventory of the use of EOtC practice in schools across Denmark conducted in 2014 [26].

Teachers participating in TEACHOUT with an EOtC class were invited to a 2-day seminar in May 2014 offering inspirational hands-on workshops on EOtC practice, networking, planning of actual EOtC teaching within their participating class, and information about TEACHOUT and their role within the study. Inspirational workshops consisted of three hands-on 90-min sessions that were all held in green areas by two teachers with at least 8 years’ experience practicing EOtC regularly. There was also a 45-min plenary introduction to the Danish EOtC inspiration and networking community and website “skoveniskolen.dk” (translated “the forest in the school”). Each of the three practical workshops used one subject as example and subjects were Math, Danish (i.e. mother tongue) and Natural Science. The workshop providers were specifically told to hand out practical tips, such as how to quickly gather children while being outdoors in an open space. Networking activities consisted of a 30-min walk and talk session and the four meals provided to teachers, which were also attended by the experienced EOtC teacher and researchers involved in the study. A voluntary e-mail and telephone list was created and handed out to participants to enable teachers to seek inspiration from one another.

In TEACHOUT, EOtC was defined as curriculum-based activities occurring outside the school’s buildings with an average duration throughout a school year of minimum 300 min per week. The EOtC practice of each participating class was monitored using an electronic questionnaire that was filled in by teachers. Data were collected between November and April of the 2014-15 school year. Teachers who provided us with the requested data received a gift certificate worth 500 Danish Kroner (~67€) in December and again at the end of the school year in June.

### Participants

Classes were recruited based on the willingness of teachers and school management to implement EOtC. To enable inclusion in TEACHOUT, classes should be within grades 3 to six, be part of a parallel class pair joining the study together, and be willing to implement a minimum average of 300 min of weekly EOtC in one or two weekly sessions throughout the school year in one class (EOtC class), but not the other (comparison class). Three-hundred weekly minutes of EOtC was chosen for it to be a substantial part of children’s school time and set the practice apart from occasionally occurring field trips. Teachers were instructed to report time spent briefing before and de-briefing after EOtC activities as EOtC.

The only exclusion criterion for children within a recruited class was known plaster allergy. The division into EOtC and comparison classes was determined by the participating schools. Recruitment was done by contacting schools known to practice EOtC based on a national survey [25] and by contacting municipalities, and through professional networks within the research group.

### Data collection

We visited classes at their respective schools. Each participant reported their birthdate, and had their height (Leicester Height Measure) and weight (OMRON BF212 Body Composition Monitor) measured. Participants had their PA measured with accelerometers, and were asked to provide diary information on certain activities they performed.

#### Physical activity measurements

We initialized Axivity AX3 accelerometers (Axivity, Newcastle, UK) to collect raw accelerometer and temperature data with a 50 Hz frequency at ±8 g bandwidth using OmGui version 1.0.0.30 (Newcastle University, UK). A female researcher taped an accelerometer directly to the skin of the lower back (right side, just above the posterior iliac crest with positive x-axis pointing downwards and negative z-axis pointing forward when standing upright) for each girl, and a male researcher for each boy. The skin was first cleaned with an alcohol wipe. Then, a 3x5cm piece of Fixomull tape (BSN Medical) was taped to the skin with a 1x2cm double-sided adhesive hair-set tape (3 M, USA) on top. The AX3 was then fixated onto the double-sided tape and covered with an 8x10cm piece of Opsite Flexifix (Smith & Nephew, UK) with rounded corners. Participants were instructed to wear the accelerometer for 10 days without removing it at any time. Data files were downloaded and stored in .cwa format using OmGui version 1.0.0.30 (Newcastle University, UK). For more information on the measurement methods, compliance and validity of accelerometer-derived PA measurements in the TEACHOUT study, see the previously published article [31].

#### Exposure to specific domains

Participants’ time spent in specific domains was logged through class timetables, class diaries and individual leisure time diaries. We gathered information at class level regarding times of practiced EOtC, recess, PE and curriculum-based classroom activities through class timetables, class diaries and the online EOtC monitoring tool. One class diary for each participating class was filled in by three children selected by the class teacher in cooperation with the teacher. The class diary requested information on specific times for any curriculum-based activities happening outside the schools’ buildings and domains in which the start and stop times differed from those stated in the class timetable. The online monitoring tool was an electronic questionnaire requiring the participating teachers to answer if the class received any EOtC on every single day throughout the school year and, if so, to provide information on the duration, location, subject taught, mode and duration of transportation to/from the location, and if the class had previously practiced EOtC on the site. In this study, we used information from the online monitoring tool to check if duration of the practiced EOtC matched between the class diary and the teacher reporting, and to compare the amount (duration) of EOtC practiced during the measured week to the classes’ average weekly amount of EOtC practice during the intervention school year. We collected information on sleep time and absence from school through individual participant diaries.

### Data processing

We calculated BMI percentiles using the method presented by Barlow and Dietz [32]. Accelerometer wear time from attachment to first following detachment was determined manually, based on visual inspection of raw accelerometry and temperature data in OmGui for each file. Start and stop wear times were added to each participant’s ID in an Excel data sheet, and wear time for each file was calculated.

Participants with seven consecutive full days of accelerometer wear time between 00:00 and 23:59 and complete information on time spent in specific domains during school hours were considered valid for inclusion in the day type analysis. The same inclusion criteria as described above for the day type analysis plus sleep times were required for a participant to be included in the specific domains analysis. For all files deemed valid for inclusion in the analyses, the 50 Hz raw accelerometer-derived data in .cwa format was converted into a binary .gt3x format compatible with the ActiLife (version v6.11.9, ActiGraph, Pensacola, FL, USA) software [33]. In the conversion, the data were resampled from 50 Hz to 30 Hz to avoid potential bias in intensity estimation calculations conducted in ActiLife [34]. The .gt3x files were transformed into .agd files with a 15-s epoch length, and were analyzed in ActiLife using Evenson [35] cut points to classify the proportion of time spent in MVPA and LPA.

For the day type analysis, we operationalized and sorted days into four categories: 1) school day with EOtC, 2) school day without EOtC and PE, 3) school day with PE, and 4) weekend day. School days with PE and EOtC could potentially have been a fifth day type, but due to low number of classes (*n* = 2) that had this type of day we did not include it. A school day with EOtC was a day with 150 min or more of curriculum-based teaching outside the school’s buildings. The 150 min cut-point was set based on what was asked of teachers participating with an EOtC class. They were asked to provide their class with an average of 300 min of EOtC per week, in one or two sessions, so the minimum expected session length was 150 min. A school day with PE was defined as a day with 45 min or more of PE. School days without EOtC and PE were those days on which we knew for certain that the class did not have 150 min or more of EOTC or 45 min or more of PE. All Saturdays and Sundays were classified as weekend days. Days with any absence from school on school days, as well as days with self-reported sickness, were excluded from the day type analysis. Sleep was included in the day analysis.

For the specific domain analysis, we included the following six categories: 1) EOtC, 2) classroom activities, 3) PE, 4) recess, 5) before/after school time, and 6) weekend. Before/after school time included the time from waking up until the start of the school day, and again from getting out of school until going to bed. Weekend included the time from waking up until going to bed on Saturdays and Sundays. Sleep was not included in the domain-specific analysis.

For the day type analysis, MVPA and LPA variables were created per day, meaning that each participant had seven unique entries, each representing a whole day in the analysis (and fewer than seven if a participant had been absent from school or sick for one or more days).

The output for the domain-specific analysis included one average proportion of MVPA and LPA per domain per participant. For example, a participant could have three separate periods of recess varying in duration on each of the five school days measured. Mean proportion LPA and MVPA of all 15-s epochs occurring within these 15 recess periods then resulted in one mean proportion of recess LPA and MVPA for him/her. To make this possible, each participant’s data files were coupled with a log diary of specific times for each specific domain and scoring was calculated in ActiLife for each domain separately, and afterwards all domains for all participants were combined in a database. Each participant’s average proportion of time spent in either MVPA or LPA in all events for a specific domain was included as one output in the data analysis.

### Statistical analyses

We used a one-way analysis of variance to test for differences in age and BMI percentile between groups in specific day types and domains. Mixed-effects residual maximum likelihood regression adjusted for the non-independence of multiple days for the same participant was used to statistically test for differences in the proportion of time spent in MVPA and LPA between specific day types as well as between specific domain types. Post-hoc multiple comparisons of marginal linear predictions, corrected for the effect of multiple testing using the Bonferroni method, were used to determine *p*-values and 95% confidence intervals for the regressions. Significance level was set to *p* < 0.05. Statistical analyses were done using Stata 14.2 (StataCorp, Texas, US).

## Results

We contacted 549 Danish primary schools to ask if they wanted to participate. Sixteen class pairs at 12 schools, with a total of 663 participants, were deemed eligible and were included in this sub-study. Data from all 663 participants in the 33 classes were pooled. The uneven number of classes is explained by one “pair” including three classes, because two of them practiced EOtC and had PE together and the third class was the comparison class. Children in 17 (*n =* 357, 54% of total children) of the 33 classes were exposed to EOtC with an average frequency of 1.3 ± 0.6 sessions during the measured week, lasting on average 212 ± 93 min per session. Table 1 shows the monthly distribution of EOtC by the participating classes and the number of children included in each analysis.

**Table 1:** Classes’ education outside the classroom-practice and number of included children during PA measurements by month

Month	Nov ‘14	Dec ‘14	Jan ‘15	Feb ‘15	Mar ‘15	Apr ‘15	Total
Classes measured	8	4	6	2	8	5	33
Classes practicing EOtC	4 (50%)	1 (25%)	5 (83%)	1 (50%)	3 (38%)	3 (60%)	17 (52%)
EOTC days	6	2	7	1	5	3	24
Minutes per EOtC day	261	113	201	210	214	200	212 ± 93
*n* in classes	174	77	121	29	168	94	663
*n* in day analysis	94	34	80	18	96	39	361
*n* in domain analysis	93	0	12	18	36	35	194

Table 2 shows participant characteristics by day type.

**Table 2:** Participant characteristics for groups by day type

Characteristic	School day with EOtC	School day without EOtC and PE	School day with PE	Weekend day
*n* (% girls)	159 (63.5%)	313 (62.6%)	291 (62.5%)	361 (61.2%)
Age, years	10.8 ± 1.2	10.8 ± 1.0	10.7 ± 0.9	10.9 ± 1.0
BMI percentile ^a^	42.0 ± 26.7	45.3 ± 28.0	46.2 ± 28.3	44.2 ± 27.5
Days	204	945	311	722

*Age and BMI percentile are reported as mean ± standard deviation*

^a^ *BMI percentile was calculated based on the definition by Barlow & Dietz* [32]

No significant differences were found in mean age or BMI percentile between samples included on different day types in the day types analysis.

Table 3 shows characteristics of groups of participants by domain.

**Table 3:** Participant characteristics for groups by domain

Characteristic	EOtC	Classroom	PE	Recess	Before/after school	Weekend
*n*, (% girls)	141 (64.5)	176 (63.1)	175 (62.9)	194 (63.4)	193 (63.7)	192 (63.5)
Age, years	10.2 ± 0.9	10.5 ± 1.0	10.1 ± 1.0	10.5 ± 1.0	10.5 ± 1.0	10.5 ± 1.0
BMI percentile^a^	47.3 ± 26.5	46.5 ± 26.8	46.5 ± 26.8	46.5 ± 26.8	46.5 ± 26.8	46.6 ± 26.9
Total events^b^	184	3525	242	1723	1764	381
Hours per person	5.1 ± 1.5	19.9 ± 3.7	1.9 ± 0.5	4.6 ± 1.3	37.6 ± 9.4	26.4 ± 2.6

*Age, BMI percentile and Hours per person are reported as mean ± standard deviation*

^a^ *BMI percentile were calculated based on the definition by Barlow & Dietz* [32]

^b^ *‘Total events’ refers to number of events in the given domain*

No significant differences were found in mean age or BMI percentile across the different domains.

**Fig. 1:**
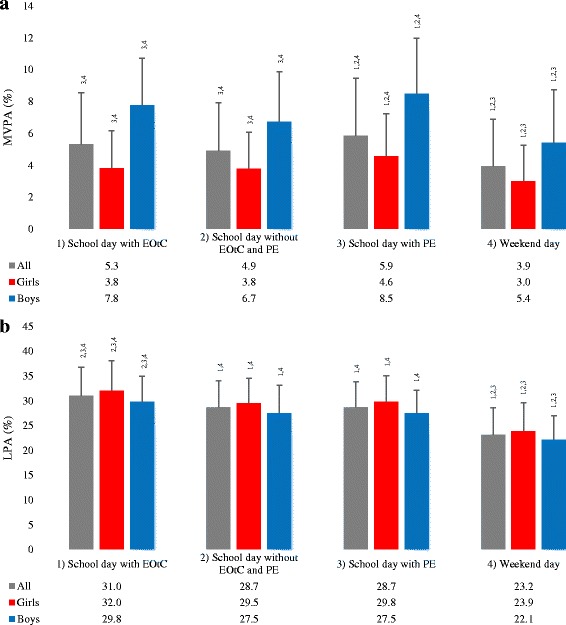
Proportion of time spent in MVPA and LPA by day type. **a** shows mean ± sd proportion of time spent in MVPA on specific day types by sample (all, girls and boys). **b** shows mean ± sd proportion of time spent in LPA on specific day types by sample (all, girls and boys). Numbers above standard deviation bars in both **a** and **b** denote significant difference (mixed-effects regression with identity link) in the proportion of time spent in the PA intensity for the sample (all, girls or boys) on a day of the given day type compared to^1^a school day with EOtC,^2^a school day without EOtC and PE,^3^a school day with PE, and^4^a weekend day

Figure 1 shows the proportion of a) MVPA and b) LPA on specific day types for the total population and for girls and boys separately. For both girls and boys, the results showed no significant differences between the proportion of MVPA between school days with EOtC and school days without EOtC and PE (both *p* = 1.000). The proportion of time spent in MVPA on school days with EOtC compared to school days with PE was 0.79% (*p* = 0.001, CI = 0.26 to 1.31) lower for girls and 1.35% (*p* = 0.003, CI = 0.32 to 2.38) for boys.

Girls spent a higher proportion of time in LPA on school days with EOtC compared to school days without EOtC and PE (2.43%, *p* < 0.001, CI = 1.21 to 3.65), school days with PE (2.18%, *p* < 0.001, CI = 0.80 to 3.56) and weekend days (8.06%, *p* < 0.001, CI = 6.85 to 9.27). For boys’ proportion of time spent in LPA on different day types, the results were similar to the girls’, with LPA being higher on school days with EOtC compared to school days without EOtC and PE (2.08%, *p* < 0.001, CI = 0.69 to 3.47), school days with PE (2.40%, *p* < 0.001, CI = 0.83 to 3.96) and weekend days (7.56%, *p* < 0.001, CI = 6.20 to 8.91).

**Fig. 2:**
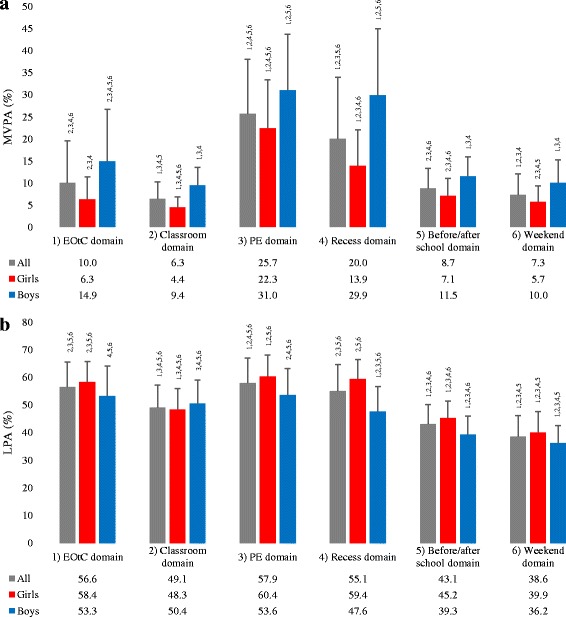
Proportion of time spent in MVPA and LPA by domain. **a** shows mean ± sd proportion of time spent in MVPA in specific domains by sample (all, girls and boys). **b** shows mean ± sd proportion of time spent in LPA in specific domains by sample (all, girls and boys). Numbers above standard deviation bars in both **a** and **b** denote significant difference (mixed-effects regression with identity link) in the proportion of time spent in the PA intensity for the sample (all, girls or boys) in a domain compared to^1^the EOtC domain,^2^the classroom domain,^3^the PE domain,^4^the recess domain,^5^the before/after school domain, and ^6^the weekend domain

Figure 2 shows the proportion of MVPA and LPA in domain-specific time for the total population and for girls and boys separately. Compared to the classroom domain, time spent in EOtC showed a 4.17% higher proportion of time in MVPA (*p* < 0.001, CI = 1.72 to 6.63) and 6.91% in LPA (*p* < 0.001, CI = 4.58 to 9.24) for all participants. For girls, no difference was found for MVPA between classroom and EOtC domains (*p* = 0.176), while the proportion of time spent in LPA was 9.76% higher in EOtC compared to the classroom domain (*p* < 0.001, CI = 7.12 to 12.41). For boys, time spent in MVPA was 7.95% higher in EOtC compared to the classroom domain (*p* < 0.001, CI = 3.00 to 12.90), while no difference was found for LPA between classroom and EOtC domains (*p* = 1.000).

Girls spent 8.66% more time in MVPA during PE compared to recess (*p* < 0.001, CI = 6.57 to 10.75), while no difference was found for boys between PE and recess (*p* = 1.000). For girls, we found no differences in the proportion of time spent in LPA between the domains of EOtC and PE (*p* = 0.147), EOtC and recess (*p* = 1.000), or PE and recess (*p* = 1.000). Also for girls, EOtC, PE and recess domains all had a higher proportion of time spent in LPA compared to the classroom domain: 9.76% for EOtC (*p* < 0.001, CI = 7.11 to 12.41), 12.03% for PE (*p* < 0.001, CI = 9.61 to 14.57), and 11.10% for recess (*p* < 0.001, CI = 8.68 to 13.52).

## Discussion

Activities in the EOtC domain were implemented instead of curriculum-based classroom activities, which should be kept in mind throughout the discussion. Generally, time spent in the EOtC domain generated more PA compared to time spent in the classroom domain; for boys in MVPA, and for girls in LPA. Girls spent around 35, and boys 30, more minutes in LPA on a school day with EOtC compared to a school day without EOtC and PE, while no difference was found in MVPA between these day types for either girls or boys. Compared to a school day with PE, girls spent 31, and boys 35, more minutes in LPA on a school day with EOtC, but 11 and 19 fewer minutes in MVPA. Both girls and boys attained a significantly lower proportion of time in both LPA and MVPA on weekend days than on any other day type. The day type analysis included only full days of valid data from 00:00 to 23:59 with the result that all reported single-day outcome measures have equal impact on daily life PA, whereas the domain-specific analysis reported varying length segments of a day, leading to the same impact of a single measure on daily life PA regardless of the duration spent in the domain by the participant. For example, PE only occurred for around 1.9 h per participant during the measured week, while recess represented 4.6 h, EOtC 5.1 h, and classroom activities 19.9 h on average.

The increase in the proportion of time spent in either LPA or MVPA in the EOtC domain compared to the classroom domain can be regarded as a positive finding when trying to achieve a more active school day. On average, each child who participated in EOtC was exposed to approximately 5 h of EOtC per week through one or two sessions. The results showed an increase in children’s weekly PA when they participated in EOtC; approximately 31 min of LPA for girls and 23 min of MVPA for boys. This also indicates a decrease in sedentary time. Holt et al. [36] reported increased PA during a school day for curriculum-based PA (and walk/run sessions) compared to other activity sessions and school days where PE and recess were the only PA opportunities provided. They reported a substantially increased frequency of implementing curriculum-based PA sessions compared to other PA sessions at their five-month follow-up evaluation compared to baseline. A tentative conclusion based on the observation by Holt et al. [36] and our results could be that curriculum-based PA integration adds in to teachers’ educational obligations and becomes more sustainable than activities that are merely added to existing teaching obligations.

If time in the classroom domain is replaced with time in the EOtC domain on a given school day, girls and boys are likely to accumulate an additional five or eight more minutes of MVPA and 24 or 6 min of LPA, respectively. Weaver et al. [13] investigated accumulated time spent in PA intensities across the school-specific domains classroom activities, PE and recess for first- to third-grade children, and found similar patterns for girls and boys as those found in this study (Fig. 2). Proportions of time spent in PA were highest in the domains PE and recess, and much lower for the classroom domain; yet more than half the average daily minutes of LPA and MVPA during school time were accumulated in the classroom domain. This is interesting, and implies the potential that lies in increasing MI in the classroom setting if it can be implemented in a way that appeals to the teacher [37]. Previous findings suggest a linear relationship between number of PA opportunities (i.e. classroom PA breaks, recess, and PE) provided and daily school time in MVPA [38]. Based on our findings, including weekly school time in the EOtC domain provides an additional opportunity for PA and thereby increases overall school time PA.

Girls engaged in lower levels of PA during recess compared to boys, which is in line with previous studies; see e.g. Bailey et al. [12]. As previously stated, girls’ proportion of time in MVPA did not differ between the EOtC and classroom domains, but was 8.7% higher in PE compared to recess, whereas for boys, MVPA during activities in the EOtC domain was 8.0% higher than in the classroom domain but did not differ between PE and recess. A recent qualitative case study of lived experiences among the least active children in an intervention aiming at increasing recess PA concluded that specific strategies, such as creating teacher-organized play activities during recess, could be more effective in increasing PA in this group [39]. One could hypothesize that those who are the least active in our study (who, in the subdivision by sex, are the girls) are achieving the highest amounts of MVPA in the instructional adult-led activities during PE, while boys to a higher degree spent time in MVPA when given the chance, whether in structured or unstructured activities. In other words, during EOtC and recess, PA was not the purpose per se, and the girls did not reach the same higher levels of MVPA as the boys did in this setting. EOtC class teachers participating in this study were not specifically instructed to increase children’s PA levels. Generally, girls’ motivation to partake in PA is both intrinsically and extrinsically driven, while boys’ motivation is more exclusively intrinsic [40]. One way to provide girls with extrinsic motivation could be through supportive environments created by teachers aiming to increase PA in places that are suited for movement, such as during EOtC, recess or PE. The implementation of future educational practices in a school setting, such as EOtC, should therefore include PA as a specific and integrated adult-structured aim, if girls are to be specifically targeted [15].

In congruence with other studies reporting domain-specific PA during a school day, such as Bailey et al. [12] and Weaver et al. [13], we highlight the importance of tailoring school-based PA interventions to target participants with certain characteristics within specific domains to increase the effectiveness of such interventions. As such, no single domain within the school setting seems able to promote PA to desired levels in a majority of children, and holistic approaches encompassing all school domains are necessary to offer sufficient opportunities to be physically active within this academic setting. The Boston Active School Day Policy [41] is an example of such a holistic approach to school-based PA promotion, aiming to increase the weekly PA of fourth and fifth graders during school hours to 150 min through targeting a combination of the domains of PE, recess and classroom activities. The intervention group was offered an 18-min greater increase in provided weekly PA opportunities compared to controls, which resulted in higher increases in MVPA and decreases in sedentary time during school hours from baseline to follow-up [41].

### Strengths and limitations

We included in our analyses only participants with at least seven full days of 24 h accelerometer wear time. Even with this strict inclusion criterion, we managed to obtain valid data from a sample of 361 participants for the day type analysis and 194 for the domain analysis. The high number of valid days without non-wear time has resulted in a high reliability and validity of the PA outcomes [31]. The included classes comprised a total of 663 children, corresponding to compliance rates of 54% in the day type analysis and 29% for the domain specific analysis. However, it should be noted that 17 of 33 classes did not provide complete time-stamped information on all school-based activities that we wanted to make distinctions between, which excluded 325 participants from this analysis before looking at the individuals PA data. Of the 338 participants in classes eligible for inclusion in the domain analyses, we obtained an inclusion rate corresponding to 57%. We did see selection bias, as the excluded participants in both analyses were older, overweight and more likely to be boys (only around 41% of eligible boys were included in the analyses), compared to the study population in general. The selection bias may also exist for PA levels as excluded children were more physically active [31]. This selection bias and subsequent generalizability issues may have become more pronounced by the strict inclusion criteria, which needs consideration when interpreting the results [42]. PA is generally lower for girls compared to boys, for overweight compared to normal weight children, and it declines with age [43, 44]. Combined with the exclusion of more children with higher PA levels, we may therefore have excluded children who accumulate both very low and very high amounts of PA compared to those included in our analyses. More in-depth information on the strengths and limitations of the accelerometer methodology used can be found in another recent paper based on the same study [31]. However, the reliability may have been somewhat compromised due to the data collection taking place during late fall, all winter and spring in Denmark. PA outcomes might have been different if we had collected data for the entire year, as weather conditions are known to impact objectively measured PA in a climate similar to the Danish one [45]. It could be hypothesized that the largely outdoor practice of EOtC [26] would make this school-based PA promotion intervention extra sensitive to weather conditions.

In the current study we observed a secular trend within educational practice in the sense that EOtC has grown as a grassroots movement in Denmark. The observed intervention was determined by the involved teachers’ way of implementing their EOtC practice and the lack of a detailed description of the implemented practice is a limitation of our study. Another limitation is that teachers’ and children’s willingness to adopt a new educational strategy, in this case to implement EOtC, could induce bias as EOtC and comparison class teachers’ characteristics, including their mentality and interests, might differ. Likewise, schools may have differing organizational opportunities to implement EOtC, and participating schools may therefore not be representative of Danish schools in general. This may compromise the generalizability of the results.

### Implications for practice, policy and research

This evaluation was part of a larger, holistic study in which academic learning, well-being and motivation for learning were all outcomes measured within the same sample of children. This cross-disciplinary and quasi-experimental design represents a novel approach to school-based PA promotion that enables us to evaluate the combined effects of EOtC on a range of outcomes that are important for practitioners and policy-makers within the school setting. Future studies should combine this cross-disciplinary data collected in TEACHOUT to gain deeper insights into the combined effects of EOtC.

We investigated EOtC because of its potential to increase PA, the rise in provision of EOtC in Danish schools, and the possibility to be included as a regular part of the curriculum-based teaching within the existing Danish school framework, without the allocation of additional funds or working hours. Findings from this study suggest that the classroom domain offers the possibility to promote PA within the school setting, but to be successful for a majority of children it is probably necessary to provide a catalogue of teaching methods that are pragmatic and well suited to teachers’ obligations. EOtC seems to be a teaching method for such a catalogue that can contribute to more active school days for children. We believe that the positive implications of EOtC on PA, while relatively small, are potentially important, as EOtC could be implemented at population level at low or no extra cost. However, we are aware that the main objective of schools is academic learning, and are therefore currently investigating and evaluating the effects of EOtC on this aspect, as well as motivation for learning, social relations, and well-being in the same group of children [16]. Future studies should combine quantitative and qualitative methods to identify professional learning components of EOtC practice that increase PA and academic learning simultaneously. An investigation specifically targeting the impact of PA on children’s school engagement and disengagement is one viable way to provide a better understanding of potential mediators between changes in PA and academic learning caused by EOtC practice.

## Conclusions

The aim of this study was to compare the effects of EOtC on children’s PA to other school-specific activities on certain day types and domains. Girls and boys spent around 36 and 33 more minutes in LPA, respectively, on a school day with EOtC compared to a school day without EOtC and PE, while no differences were found in MVPA for the two groups. Compared to a school day with PE, girls spent 32, and boys 33, more minutes in LPA on a school day with EOtC, but 11 and 19 fewer minutes in MVPA. EOtC activities generated more PA for children compared to classroom activities during school hours; for boys this was of moderate or higher intensity while for girls it was of lower intensity.

In the evaluated intervention, EOtC was implemented in the existing Danish school system without the allocation of extra resources, and was practiced regularly over a full school year. PA was measured in a large sample with high validity and reliability. Therefore, positive effects on PA seem plausible with implementation at population level. Future research should evaluate how EOtC and its effects on PA relate to other important school outcomes, such as academic learning, and investigate what characterizes good EOtC practice in order to guide policy and practice.

## Abbreviations

EOtC: Education outside the classroom
LPA: Light physical activity
MI: Movement integration
MVPA: Moderate-to-vigorous physical activity
PA: Physical activity
PE: Physical education
